# Screening of recombinant proteins as antigens in indirect ELISA for diagnosis of bovine tuberculosis

**DOI:** 10.1186/2193-1801-1-77

**Published:** 2012-12-22

**Authors:** Ingrid IF Souza, Elaine SP Melo, Carlos AN Ramos, Thaís A Farias, Ana Luiza AR Osório, Klaudia SG Jorge, Carlos ES Vidal, Altino S Silva, Márcio R Silva, Aiesca O Pellegrin, Flábio R Araújo

**Affiliations:** 1 Pós Graduação em Ciência Animal, Universidade Federal de Mato Grosso do Sul, Campo Grande, MS Brazil; 2 DTI CNPq grant holder, Embrapa Gado de Corte, Campo Grande, MS Brazil; 3 Pós Graduação em Medicina Veterinária, Universidade Federal de Santa Maria, Santa Maria, RS Brazil; 4 Instituto de Defesa Agropecuária e Florestal do Espírito Santo, Vitória, ES Brazil; 5 Embrapa Gado de Leite, Juiz de Fora, MG Brazil; 6 Embrapa Pantanal, Corumbá, MS Brazil; 7 Embrapa Gado de Corte, Campo Grande, MS 79106550 Brazil

**Keywords:** Serology, ELISA, Recombinant proteins, *Mycobacterium bovis*, Cattle

## Abstract

Bovine tuberculosis is an important infectious disease caused by *Mycobacterium bovis*, which is responsible for considerable economic losses. This disease constitutes a serious public health problem. Control programs in most countries, including Brazil, are based on the identification and slaughter of infected animals, as defined by the skin tuberculin test, which has its constraints. In the present study, the recombinant proteins CFP-10, ESAT-6, Mb0143, MPB83, PE5, PE13, TB10.4, TB15.3 and a chimera of ESAT-6/MPB70/MPB83 (fusion protein) were tested as ELISA antigens for the diagnosis of bovine tuberculosis. The proteins were produced in *Escherichia coli,* purified and tested in ELISAs with sera from 126 cattle having tested negative in the comparative intradermal tuberculin test (CITT) and 107 sera from cattle having tested positive in the CITT. Also, 236 sera from two BTB-free beef cattle herds were tested. Among the proteins tested, only the ESAT-6/MPB70/MPB83 chimera demonstrated satisfactory agreement with the CITT (*kappa* index: 0.688), reflecting in 83.2% sensitivity and 86.5% specificity. The ELISA absorbances of the cattle sera from BTB-free herds showed similar levels to those of CITT positive cattle, probably as the result of successive skin tuberculinizations to define the BTB-free status of the herds. However, the ELISA with the ESAT-6/MPB70/MPB83 chimera was useful to discriminate BTB positive and negative cattle in herds prior to the tuberculin skin test.

## Background

Bovine tuberculosis is a chronic infectious disease caused by *Mycobacterium bovis,* which affects cattle (Wobeser 2009), other domesticated species, wild animals and humans (Gutierrez et al. 1998; Michel et al. 2011). This disease causes economic losses in livestock farming and poses a health risk to the population that consumes products of animal origin (Lilebaum et al. 2001; Bennett and Cooke 2006).

The detection of tuberculosis in cattle is based mainly on the measurement of delayed hypersensitivity following the intradermal injection of *M. bovis* antigens, which are usually purified protein derivatives (Whelan et al. 2011). Despite the effectiveness of diagnostic approaches based on the detection of the cellular immune response to *M. bovis* antigens, skin tests must be conducted *in vivo*, which is difficult in large-scale epidemiological surveys and retrospective epidemiological analyses (Menzies 1999; Liu et al. 2007). Moreover, a number of chronically infected animals may go undetected even after skin and IFN-γ tests, which places herds at risk for the bovine-to-bovine spread of infection.

Antibody responses to *M. bovis* are positively correlated with the mycobacterial-elicited pathology and antigen burden. Thus, serological tests may increase the degree of detection of animals infected with *M. bovis* (Whelan et al. 2011). Serological tests may also constitute an alternative for screening herds for *M. bovis* infection as well as retrospectively testing sera from control programs for other diseases, such as bovine brucellosis.

A limited number of antigens have been tested in immunoassays for the diagnosis of bovine tuberculosis. Antigens such as ESAT-6, MPB70 and MPB83 have been extensively evaluated in enzyme-linked immunosorbent assay (ELISA) (Buddle et al. 1995; Amadori et al. 2002; Farias et al. 2012), however, its sensitivity and specificity are relatively low. Thus, the search for new antigens for serological diagnosis of bovine tuberculosis is required.

The aim of the present study was to test the recombinant proteins CFP-10, ESAT-6, Mb0143, MPB83, PE5, PE13, TB10.4, TB15.3 and a chimera of ESAT-6/MPB70/MP83 peptides as ELISA antigens for the diagnosis of bovine tuberculosis. CFP-10, ESAT-6 and MPB83, as a chimera of ESAT-6/MPB70/MP83 have been previously tested as an antigen (Amadori et al. 2002; Liu et al. 2007), however, Mb0143, PE5, PE13, TB10.4 and TB15.3 are being evaluated for the first time. The selection of these proteins was based on the localization of the protein in cell wall of the *Mycobacerium bovis*, such as PE5 and PE13 (Garnier et al. 2003; Aagaard et al. 2006), or because they are secreted proteins as TB10.4 and TB15.3 (Mattow et al. 2003). Mb0143 is a conserved hypothetical protein and has been evaluated in IFN-γ assay (Meikle et al. 2009).

## Results

### Production of recombinant proteins

The fragments corresponding to the coding regions of genes *cfp-10, esat-6, mb0143, mpb83, pe5, pe13, tb10.4,* and *tb15.3* were amplified by PCR, generating amplicons of 300, 288, 459, 604, 270, 219, 231 and 408 base pairs, respectively.

Gene expressions were determined through electrophoresis in SDS-PAGE, which revealed the recombinant proteins CFP-10, chimera of ESAT-6/MPB70/MPB83, ESAT-6, MPB83, MB0143, PE5, PE13, TB10.4 and TB15.3 to have 13.6, 35, 12.9, 20.5, 28.5, 17.5, 12.5, 11.6 and 17.5 KDa, respectively. These findings are consistent with the *in silico* predicted molecular masses analyzed using the EdiSeq of the DNASTAR program, indicating that the proteins were not post-translationally modified. In Western blotting, all recombinant proteins were recognized by the anti-6x-histidine antibody, thereby confirming gene expression.

### ELISAs

Table 1 displays the optimal dilutions of sera and antigen concentrations. To evaluate the accuracy of the ELISAs, serum samples were obtained from cattle tested positive in the CITT and from cattle tested negative in the CITT from different states of Brazil. The technicians were blinded to the sample sets being tested and corresponding skin test status.

**Table 1:** **Optimal dilutions of sera and recombinant proteins of*Mycobacterium bovis*for ELISA**

ELISA	Optimal dilutions	
	Serum	Recombinant proteins (final concentration μg/ml)
CFP-10	1: 600	1: 10,000 (0.1)
Chimera ^*^	1: 600	1: 5000 (0.2)
ESAT-6	1: 600	1: 5000 (0.2)
Mb0143	1: 600	1: 10,000 (0.1)
MPB83	1: 600	1: 10,000 (0.1)
PE13	1: 600	1: 500 (2.0)
PE5	1: 400	1: 500 (2.0)
TB10.4	1: 400	1: 2000 (0.5)
TB15.3	1: 400	1: 5000 (0.2)

* Chimera – fusion of MPB70, MPB83 and ESAT-6 fragments.

**Figure 1:**
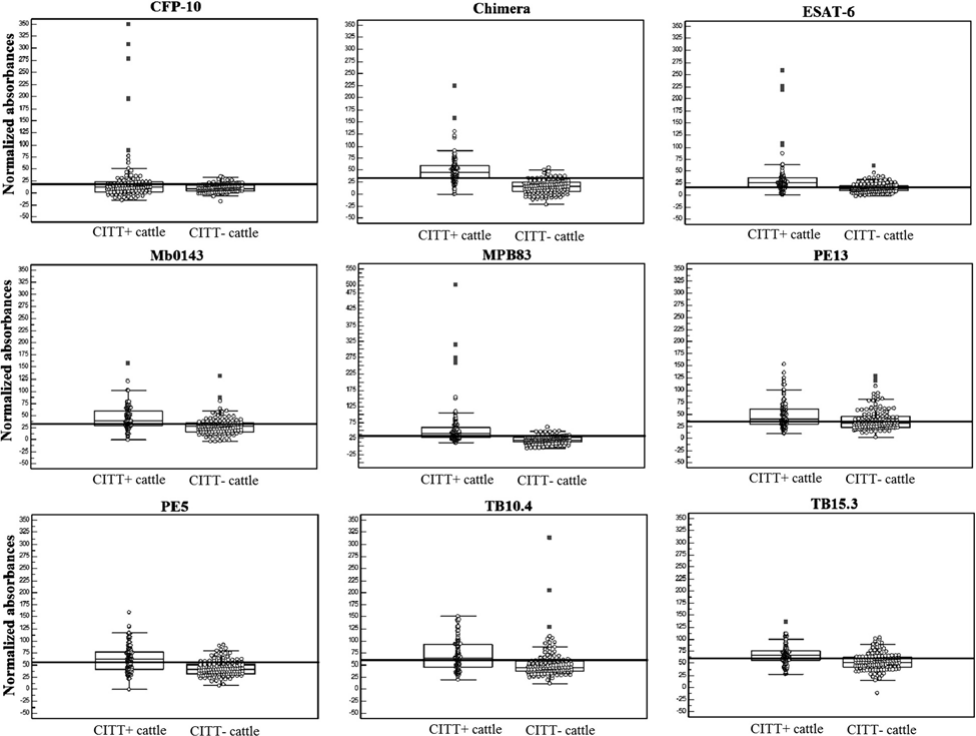
**Distributions of normalized absorbances of ELISAs with recombinant proteins of*Mycobacterium bovis.***

Figure 1 displays the normalized ELISA absorbances. Results are expressed as relative sensitivity or relative specificity taking CITT as the reference test. Relative sensitivity ranged from 28.0% to 83.2% and relative specificity ranged from 58.7% to 92.9%. The highest relative specificity (92.9%) of the ELISA with CFP-10 was in detriment to the relative sensitivity of the test (28.0%).

Regarding the *kappa* test, only ELISA with the chimera of the ESAT-6/MPB70/MPB83 peptides demonstrated adequate agreement with the CITT (*kappa* index: 0.688; confidence interval: 0.595-0.782). ELISAs with ESAT-6, MPB83 and PE5 exhibited fair agreement with the CITT (*kappa* index: 0.447, 0.554 and 0.404, respectively) and the other ELISAs exhibited minimal agreement with the CITT (*kappa* index: < 0.4).

**Figure 2:**
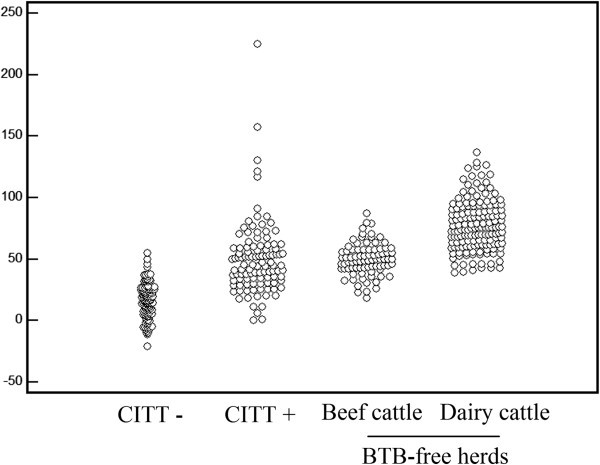
**Dot-plot showing normalized ELISA absorbance of sera from CITT negative, CITT positive cattle, BTB-free beef cattle and dairy cattle herds.** The chimera antigen (MPB70/MPB83/ESAT-6) alone was used as a model in this figure, as it provided better differentiation between positive or negative cattle for tuberculosis.

The normalized ELISA absorbances of the cattle sera from BTB-free herds showed similar levels to those of CITT positive cattle (Figure 2).

## Discussion

The performance of the ELISA with the ESAT-6/MPB70/MPB83 chimera was superior to that of ESAT-6 or MPB83 individually. It was not possible to analyze MPB70 individually as an antigen due to problems with the purification of the recombinant protein.

The kinetics of the antibody responses to different *M. bovis* antigens is variable upon infection. Anti-MPB83 antibodies can be detected relatively early, typically around four weeks post-infection (Waters et al. 2006). Anti-ESAT-6 antibodies can be detected 12 weeks after experimental infection (Lyashchenko et al. 1998). In contrast, antibodies for MBP70 usually develop 18 to 22 months after experimental infection (Harboe et al. 1990; Fifis et al. 1992). Thus, chimeras containing important epitopes of major proteins of *M. bovis* are expected to be able to detect antibodies from cattle in different stages of infection, thereby increasing the diagnostic coverage. Similar results are reported in a chemiluminescent assay with multiple antigens, in which sensitivity and specificity were superior to those of individual antigens, including ESAT-6 and MPB83 (Whelan et al. 2008). The choice for the production of a chimera of antigens instead of a cocktail of individual antigens is justified by the facility of a single expression and protein purification, with lower costs and no issues regarding the ratio of various antigens.

The cutoff values in this study were determined using ROC analysis, resulting, in the case of ELISA with the ESAT-6/MPB70/MPB83 chimera, in a relative sensitivity of 83.2% and an a relative specificity of 86.5%, taking CITT as the reference test. If we take a practical relative specificity of 95%, the relative sensitivity would be 67.3%, which is similar to those found with another chimera using the same antigens (69.4% sensitivity and 96% specificity) reported in a previous study (Liu et al. 2007). ELISA with a blend of MPB70 and MPB83 has also demonstrated good results (63% sensitivity and 98% specificity) (Waters et al. 2011). These findings strongly support the use of multiple antigens for the detection of cattle infected with *M. bovis*.

Besides the antigens that are traditionally used for serology (ESAT-6, MPB70 and MPB83), others that have demonstrated promising results in cellular assays were tested for serology, such as CFP-10, Mb0143, PE13, TB10.4 and TB15.3 (Aagaard et al. 2003; Aagaard et al. 2006). Among these antigens, only ELISA with PE5 demonstrated fair agreement with the CITT. Our group also found promising results with PE5 in intradermal tests with cattle experimentally immunized with the inactivated AN5 strain of *M. bovis* (data not shown). The potential of this antigen in chimera constructs will be evaluated in future studies using ELISA for *M. bovis* antibodies.

The similar responses of two BTB-free herds and CITT positive cattle suggest that the repeated tuberculin test, which is necessary for the establishment of the BTB-free status of the herd, may act as a booster to antibody production, as described in other studies for BTB (Harboe et al. 1990, Monaghan et al. 1994) and for bovine paratuberculosis (Varges et al. 2009). Other possibility is the sensitization of the BTB-free animals with environmental mycobacteria, and cross-reactions with the antigens, but with the herd of Embrapa Beef Cattle, which was tested with tuberculin only on the same day that serum samples were obtained, the discrimination was clearly higher. For this reason, ELISA is a useful tool for the identification of BTB positive herds prior to the tuberculin skin test.

As in many countries, the diagnosis of bovine tuberculosis in Brazil is based on routine testing with the intradermal tuberculin test. Skin tests require containment of the animals on two occasions with a 72-h interval for measuring the thickness of the skin fold (Brasil 2004). Management is hampered in herds raised extensively or on farms without adequate facilities, which is a common situation in developing countries. This difficulty is probably one of the main reasons for why epidemiological surveys of tuberculosis planned in the eradication program in Brazil are in a less advanced stage than those for brucellosis.

An ancillary antibody-based assay, such as ELISA with the ESAT-6/MPB70/MPB83 chimera, offers the possibility of identifying BTB positive herds using sera collected for other epidemiological studies, such as for brucellosis, prior to tuberculin skin tests, thereby increasing the diagnostic coverage. This is another useful aspect in the possible identification of cattle in advanced stages of tuberculosis with false-negative results on the skin test due to anergy (Whelan et al. 2011).

## Materials and methods

### Antigens

*Mycobacterium bovis* DNA (AN5 strain) was purified from Stonebrink’s medium cultures using a commercial kit (DNeasy Blood & Tissue kit, Qiagen). The primers were designed with the *PrimerSelect* program (DNAStar) to amplify the *cfp-10, esat-6, mb0143, mpb83, pe5, tb10.4* and *tb15.3* genes (Table 2). Polymerase chain reactions (PCR) were carried out in a volume of 25 μl, containing 20 mM of Tris, pH 8.4, 50 mM of KCl, 1.5 mM of MgCl_2_, 250 μM of each dNTP, 100 ηg of each primer, 0.2 U of *Taq* DNA polymerase (Invitrogen) and a 50 ηg of *M. bovis* DNA. The amplification protocols were performed based on the characteristics of each pair of primers.

**Table 2:** **Primers for amplification of gene fragments coding for*Mycobactyerium bovis*antigens**

Gene	DNA sequence
*cfp-10*	5’ GCAGACATGAAGACCGATGCCGCTACC 3’
	5’ TCACAAGCCCATTTGCGAGGACAGC 3’
*esat-6*	5’ ATGACAGAGCAGCAGTGGAATTTC 3’
	5’ CTATGCGAACATCCCAGTGAC 3’
*mb0143*	5’ GCTTCGGAGTTCTCCCGTGCTGAA 3’
	5’ CTCGTCGAGGGTGCCCAACTCCT 3’
*mpb83*	5’ ATGATCAACGTTCAGGCCA 3’
	3’ GAACTCCGCCACATACCAAA 3’
*pe13*	5’ TCTTTCGTGATGGCATACCC 3’
	5’ GACTTCAGTGGCCGAA 3’
*pe5*	5’ ATGACGTTGCGAGTGGTTC 3’
	5’ TCAGCCGCCCACGAC 3’
*tb10.4*	5’ ATGTACAACTACCCCGCGAT 3’
	5’ CATGGTGTTGGCTTCATGG 3’
*tb15.3*	5’ AGCGCCTATAAGACCGTGGTGGTA 3’
	5’ CTTGGCCCGGCGTGACACATTGG 3’

Following amplification, the genes were initially cloned in *pGEM-T Easy* (Promega) plasmid, following the manufacturer’s instructions. Cloned genes were sequenced in both directions using the BigDye Terminator v.3.1 kit (Applied Biosystems). After digestion with *Eco*RI, the genes were subcloned in *pET47-b* (Novagen), except *tb10.4*, which was cloned in *pET28-a* (Novagen), and *mpb83*, which was cloned in *pRSET-C* (Invitrogen).

An 846-nucleotide synthetic chimera was constructed (Genone) with DNA coding sequences for the hydrophilic domains of ESAT-6, MPB70 and MPB83 and cloned in the *Eco*RI site of *pET47-b*. This chimera was constructed with nucleotides 18693–19025 of *mpb70* (BX248344.1, fragment 11/14), 15664–15966 of *mpb83* (BX248344.1, fragment 11/14) and 221989–222198 of *esat-6* (BX248341.1, fragment 14/14).

The *Escherichia coli* Rosetta strain was used as the host cells for all DNA constructs. The induction of gene expression was performed using 1 mM of IPTG in 500 ml of LB broth with 50 μg/ml of chloramphenicol and 30 μg/ml of kanamycin for *pET47-b* and *pET28-a* or 50 μg/ml of chloramphenicol and 100 μg/ml of ampicillin for *pRSET-C* at 37°C for 4 h at 250 rpm. Gene expression was confirmed using SDS-PAGE and Western blotting with the anti-6x-histidine monoclonal antibody (Sigma).

Recombinant proteins were solubilized with 6 M of HCl-guanidine and purified using the His-Trap HP agarose-nickel resin (GE Healthcare), following the manufacturer’s instructions. Recombinant proteins were dialyzed with PBS at 4°C for 48 h and concentrations were determined by comparisons with known concentrations of bovine serum albumin in SDS-polyacrylamide gel (SDS-PAGE), using the LabImage v.3.3.2 program (Loccus).

As the proteins became insoluble after dialysis, solubilization with 2% SDS was conducted as described elsewhere (Lechtzier et al. 2002).

### Evaluation of ELISAs

Optimal dilutions of recombinant proteins, sera and conjugate were determined through the analysis of four sera from cattle having tested positive in the comparative intradermal tuberculin test (CITT) and four sera from cattle having tested negative in the CITT (Table 1). The CITT was carried out and interpreted according to the technical regulation of the National Program of Control and Eradication of Brucellosis and Tuberculosis of the Brazilian Ministry of Agriculture (Brasil 2004).

Sera from 107 cattle having tested positive in the CITT from Caarapó, state of Mato Grosso do Sul, Itapemirim, state of Espírito Santo and Toropi, Tapera, São Martinho da Serra and Santa Maria, state of Rio Grande do Sul, Brazil, were evaluated in the ELISAs. Sera were obtained in non-specific time-point post skin tests.

Sera from 126 cattle having tested negative in the CITT from Campo Grande, state of Mato Grosso do Sul, Brazil, were also tested in the ELISAs. The CITT negative animals were from a herd with no history of bovine tuberculosis (BTB) of Embrapa Beef Cattle. Cattle from this research center are often slaughtered in official abattoirs, and there were no reports of BTB positive animals. Blood was collected for sera on the same day that the CIIT was carried out, to avoid interference from the tuberculin test on the immune status of the animals.

Among 126 animals having tested negative in the CITT, 51 (40.5%) were slaughtered and no lesions suggestive of BTB were found during meat inspection at an official slaughterhouse. All CITT positive animals were from herds with outbreaks of BTB, as evidenced by the presence of suggestive lesions during slaughter, the growth of acid-fast bacilli and PCR with primers for the *M. tuberculosis* complex (Rodriguez et al. 1995).

Moreover, 154 sera from a BTB-free beef cattle herd and 82 sera from a BTB-free dairy cattle herd were tested. The BTB-free status of the herds was established based on CITT, in compliance with the technical norms of the National Program for the Control and Eradication of Brucellosis and Tuberculosis of the Brazilian Ministry of Agriculture. Briefly, a herd is considered free of BTB after three consecutive negative tuberculin tests, with an interval of 90–120 days between the first and second tests, and an interval of 180–240 days between the second and third test (Brasil 2004).

Costar 3590 polystyrene 96-well plates (Corning) were adsorbed with each antigen in carbonate-bicarbonate buffer, pH 9.6, for 60 min at 37°C. The plates were then blocked with 100 μl/well of phosphate buffer saline with 0.1% Tween 20 (PBST) with 5% skim milk for 60 min at 37°C. After five washes with PBST, 100 μl/well of the control and test sera diluted in PBST with 2% skim milk were incubated for 60 min at 37°C. The plates were washed five times with PBST. Next, 100 μl of monoclonal antibody anti-bovine IgG (heavy chain) horseradish peroxidase conjugate (Sigma, A5295) (dilution: 1:10,000 in PBST) were added to each well. The plates were incubated for 30 min at 37°C, washed five times and 50 μl/well of chromogen/substrate Fast OPD (Sigma, P9187) were added to each well. The reactions were stopped with 2.5 N of H_2_SO_4_ and the results were read on an EL-800 ELISA reader (Bio-Tek) with a 490 ηm filter.

To minimize the effect of intra-assay and inter-assay variations, ELISA absorbances were normalized based on the methodology described by Ramanakumar et al. (2010).

### Statistical analysis

ELISA cutoff points were determined by Receiver Operating Characteristic analysis (Metz 1978; Zweig and Campbell 1993), using the MEDCALC .10.3.0.0 software program. Agreement between CITT and ELIZA was assessed by kappa index according to Ansari-Lari (2005).

## Purpose of the work

We wanted to develop an ancillary diagnostic test to bovine tuberculosis, based on ELISA with recombinant proteins.
